# The unit size effect on chocolate consumption: How to make consumers eat less? (The unit size effect on chocolate consumption)

**DOI:** 10.1016/j.heliyon.2024.e41139

**Published:** 2024-12-13

**Authors:** Enikő Kontor, Mihály Soós, Nikolett Balsa-Budai, Sándor Kovács, Zoltán Szakály

**Affiliations:** a University of Debrecen, Faculty of Economics and Business, Institute of Marketing and Commerce, H-4032, Debrecen, Böszörményi Str. 138, Hungary; b University of Debrecen, Faculty of Economics and Business, Institute of Sectorial Economics and Methodology, H-4032, Debrecen, Böszörményi Str. 138, Hungary

**Keywords:** Nudge, Size enhancement, Package, Consumer perception, Chocolate

## Abstract

Understanding the impact of environmental stimuli, such as nudges, on consumption behavior is crucial for developing effective dietary interventions. This study investigates the unit size effect, a behaviourally-oriented nudge, on chocolate consumption. In particular, it examines how different unit sizes and the presence or absence of packaging influence the quantity of chocolate consumed and the perceived energy intake in grams and calories. The research contributes to the theoretical framework of nudge theory, particularly the concept of unit bias, which suggests that larger units lead to higher consumption volumes. The novelty of this paper lies in its exploration of the combined effects of unit size and packaging and healthy lifestyle on consumption behavior, a relatively under-researched area. The methodology involved an experiment with four focus groups, differentiated by health-consciousness, who were provided with chocolate in varying unit sizes and packaging conditions. The data from the experiments were subjected to a three-way repeated measures ANOVA (RM-ANOVA) model. The results indicated that larger unit sizes significantly increase consumption compared to smaller units, regardless of packaging. This demonstrates that unit size has a greater impact on consumption than packaging, reinforcing the power of unit bias. The health-conscious participants consumed a markedly lower quantity of packed chocolates and exhibited a tendency to significantly overestimate the amount consumed, suggesting that those who are health-conscious may be more conscious of their diet. The findings support the effectiveness of behaviourally-oriented nudge marketing tools in influencing consumption behavior without altering consumer knowledge or emotions.

## 1 Introduction

Over the past decades, diseases of civilization have emerged as one of the leading causes of death globally [1]. Our dietary habits, which fail to adapt to our altered lifestyle, appear to play a significant role in this. Consequently, dietary habits and nutritional behaviour represent a highly active field of research. A particularly important aspect of nutritional behaviour is the fact that not all the factors influencing our dietary decisions are conscious; rationality often takes second place. A number of factors exert an influence on our perception rather than our conscious mind. There are two strategies available for changing people's dietary habits. The first is traditional, involving behavioural change. These strategies are primarily interventions set out in policies by the state, the sector, or civil society organisations (education, economic incentives, prohibition). The second group of strategies implies a novel approach to behavioural change, which are typically implemented at the company level or at the level of individuals [2].

The foundations of the nudge approach were established by Thaler and Sunstein (2008) [3]. They posit that changing consumers' habits is impeded by a number of challenges and necessitates a systematic approach, whereas altering certain elements of the environment where consumer choices are made can be more expedient and more efficacious. In addition, the authors introduced the concept of choice architecture, which posits that when designing a choice environment, architects should also consider the traps and biases described in behavioural decision theory. This suggests that people are not always capable of making rational decisions due to their bounded cognitive ability and the complexity of their environment. Consequently, in Thaler and Sunstein's interpretation (2008), the term ‘nudge’ is used to describe the manner in which decision-makers are influenced and steered towards a particular direction [3]. Even though the nudge steers the decision maker towards a pre-defined decision, their freedom of choice is still preserved [4]. The term ‘nudge’ is used to describe any aspect of decision-making that can alter people's behaviour in a predictable way without prohibiting any choice or employing any economic incentive [3].

Several versions of nudges supporting conscious and healthy nutrition have been identified in the literature, and they have also been grouped into several categories [[5, 6, 7, 8, 9, 10], among others]. Cadario and Chandon (2020) follow the classic tripartite classification of activities (cognition, affect, behaviour), and describe a total of seven types of healthy nutritional nudges grouped in three categories: a) Cognitively oriented interventions (descriptive nutritional labeling, evaluative nutritional labeling, visibility enhancements), which aim to influence and change consumers' knowledge and information; b) Affectively oriented interventions (hedonic enhancements, healthy eating calls), which influence consumers' feelings without changing their level of knowledge; c) Behaviourally oriented interventions (convenience enhancements, size enhancements), which influence consumers’ behavior (e.g. their motor responses) without changing their level of knowledge or feelings [11].

The aim of this study is to place more emphasis on behaviourally oriented interventions. The second sub-category of behaviourally oriented interventions is that of size enhancements, which can be achieved by changing the size of portions, the size of (diameter) of plates or glasses, or by differentiating the types of pre-portioned and pre-plated foods based on their nutritional value. Studies have demonstrated that modifications to the size of plates, glasses, and portions are most effective when consumers are minimally attentive to these changes in size and portion [12]. This contrasts with the case of cognitively oriented interventions, where visual attention is a crucial factor. Conversely, the term “unconsciousness” is of significant benefit in this context [10,13,14]. While cognitively oriented interventions and affectively oriented interventions can influence food decisions through sight and hearing, behaviourally oriented interventions exert their effect through physical interactions [11]. Furthermore, this category warrants particular attention, given that Cadario and Chandon's meta-analysis (2020) demonstrated that behaviourally oriented interventions were the most effective in reducing calorie intake [11].

However, despite extensive research, we still have limited knowledge about how subtle environmental changes can influence dietary choices. Particularly in the context of behavioural interventions, this needs further investigation. To address the identified research gap, the study focuses on these types of interventions, including specifically size enhancement. The research question can therefore be formulated as follows:

• What impact do different unit sizes and the presence or absence of packaging have on the amount of chocolate consumed and perceived energy intake, especially concerning health consciousness?

There are many ways in which this paper contributes to existing theories and approaches. Firstly, it addresses the relatively underexplored area of unconscious influences on dietary behaviour, contrasting with the more commonly studied conscious decision-making factors. The present study does not examine conscious decision-making; rather, it considers how unit size and packaging influence consumption without altering consumers' knowledge or emotions. This fundamental difference highlights the novelty of the research. The investigation of unit size and packaging focuses on aspects of nudge theory that operate automatically, outside of conscious control, affecting consumer behaviour.

Secondly, while a substantial corpus of literature on nudge theory in food consumption has concentrated on the effects of portion sizes [15, 16, 17, 18, 19, 20, 21, 22, 23, 24, 25] and product labelling [11], there has been a paucity of studies investigating the combined impact of unit size and packaging on actual consumption behaviour, particularly in the context of indulgent foods such as chocolate.

Thirdly, it applies a specific nudge strategy (size enhancement) within a unique context, examining its effects on health-conscious and less health-conscious consumers. Based on consumer characteristics, this context-specific research provides new insights into the different effects of nudges.

Finally, as seen above, findings suggest that behaviourally oriented interventions, particularly size enhancements, are more effective in reducing unhealthy eating habits than in promoting healthy eating. This provides practical implications for the design of effective dietary interventions.

By reviewing the relevant literature, the following sections examine the impact of portion size, unit size and packaging on consumption and perceived energy intake. It then describes the conditions under which the focus group interviews were conducted and the statistical methods used. The results are compared with those of similar international studies. The paper concludes with a discussion of the conclusions, implications and limitations of the study.

## 2 Impact of unit size and packaging on consumption

How much we consume at a time is influenced in two different ways by our immediate environment. First, the environment (e.g. package size, food selection) provides us with subtle hints on how much food is typical, reasonable or normal to eat during a single eating occasion. This phenomenon is known as the development of consumption norms. Secondly, these environmental cues can make us unconsciously (instinctively) ignore the internal signs of hunger and accept external cues as rules of thumb to gauge when it is appropriate to stop eating (e.g. clean our plate no matter how full we feel) (reduced ability to monitor food intake) [15, 16, 17].

As previously stated, consumers exhibit a lack of interest in decisions regarding food selection. They devote minimal energy to collecting and evaluating related information. This is why consumers are prone to rely on so-called consumption norms when deciding on the amount of food to eat. The availability of larger packages in grocery stores, larger servings in restaurants, and larger kitchenware and portions all subtly influence consumers to develop a consumption norm that makes them believe “this is the appropriate amount, the normal portion for us” regardless of their hunger levels or the number of calories they should consume [15]. The development of consumption norms is influenced by external environmental cues, and it typically occurs as an automatic (unconscious) process outside of conscious awareness and conscious decision-making [16,17].

One of the key factors that contributes to overeating is the growth of food portions. This growth has a fundamental effect on how much people usually consume, as we tend to eat more if we receive a larger portion. This phenomenon is referred to as the “portion size effect” and has been the subject of numerous studies [17, 18, 19, 20, 21, 22, 23, 24, 25].

The reason behind the urge to eat the whole serving or portion is manifold. Firstly, in childhood we are taught the unwritten rule and cultural norm that tells us we “must not leave any uneaten food on our plate” [17,26]. Secondly, we are also prone to “perform”, and this pressure leads us to eat up everything we are offered. Thirdly, as consumers, we are driven to obtain the greatest value for our money. Finally, as larger portions are considered to be the consumption unit, and people tend to eat a complete unit, control over eating decreases [22]. This phenomenon is referred to as unit bias, which describes the tendency of individuals to consume a complete unit, regardless of its size [25,26]. It is essential to distinguish between portion size and unit size. Studies on portion size alter the amount of food served or packaged, whereas studies on unit size (or the size of food items) maintain the portion size and divide the food into several units [[17, 27, 28], among others].

In terms of unit size, the numerosity heuristic – a kind of decision bias – is particularly important. To put it simply, this bias predicts that people tend to infer greater quantity from higher numerosity (a higher number of units). Consequently, when individuals perceive that they have consumed a greater number of smaller-sized food items, they may conclude that they have consumed sufficient calories and subsequently cease eating at an earlier point in time compared to instances in which they consume a single larger-sized food item [22]. The results of an experiment conducted by Wu et al. (2023) demonstrated that the number of units of chocolate influenced the perception of both the total portion size and the unit size [29]. Vandenbroele et al. (2019) additionally identified further distinctions between unit size and unit number [28]. The researchers assumed that the numerosity heuristic implies that people perceive the quantity of food as greater when the number of units is increased compared to situations in which the size of the units is increased. In their study, Vandenbroele et al. (2019) demonstrated that the portion size effect is stronger for increases in unit size than unit number [28]. This implies that relative consumption is greater when portions consist of increased unit sizes than increased unit numbers. In their work, Joye, Bruyneel and Fennis (2021) identified the existence of the “Gestalt-bias” effect in the case of the unit bias and the portion size effect. They also found that the subjective construction of food units forms a new perceptual unit [30].

The packaging of food products also plays a significant role in influencing consumer behaviour. Studies have demonstrated that the absence of packaging can result in increased food intake due to a reduction in self-regulation [17,31]. For instance, unwrapped chocolates are more likely to be consumed in larger quantities than wrapped ones, as the act of unwrapping provides a moment for self-regulation and reconsideration [32]. Previously, research had examined whether the individual packaging of food units could also play a role in how much is consumed [31]. The opening of small units of food requires additional effort, which may influence consumption compared to cases where one larger unit is eaten. Cheema and Soman (2008) demonstrated that smaller units lead to a decrease in consumption. According to them, in the case of smaller units, the decision whether to continue eating will receive extra attention, which makes eating a less automatic activity [31]. In addition to more deliberative decision opportunities, empty wrappers may also indicate that an individual has eaten enough. This effect is particularly pronounced in environments where individuals are exposed to continuous eating opportunities, such as parties or workspaces.

The perception of energy intake is a critical factor in dietary regulation. In their review, Chu, Tang and Hetherington (2021) demonstrated that modifying the characteristics of packaging, such as its sizes, can effectively reduce food intake [33]. Tal, Gvili and Amar (2021) also found that unit sizes and packaging can distort individuals' perceptions of how much they have consumed [34]. Participants often underestimate their caloric intake when consuming larger units or unpackaged foods, leading to a higher likelihood of overeating. This suggests that environmental cues, such as unit size and packaging, can override personal health intentions. However, Woolley and Liu (2021), who investigated the method of calorie estimation in their study, stated that it is possible that calorie estimation responds differently to the type of stimulus that is processed first (e.g., healthiness) and the quantitative information that is processed second (e.g., portion size) [35]. The findings of the Liu et al.‘s (2018) study yielded comparable results [36].

Much research has been conducted on the effects of unit bias, the presence or absence of packaging, and health considerations on consumption, although it is noticeable that the effect of unit size has been relatively understudied compared to the more commonly known portion size effect. Research indicates that larger units generally lead to increased consumption, particularly of unhealthy foods such as candies and cookies. Studies by Marchiori et al. (2011, 2012) demonstrated that reducing the unit size of candies and cookies significantly decreased intake by participants, highlighting the role of unit size in moderating consumption habits [37,38]. Similarly, Marchiori, Papies and Klein (2014) found that larger pieces of chocolate led participants to consume more, as these larger units were perceived as appropriate servings [39].

Further supporting these findings, Wansink et al. (2011) observed that offering crackers in smaller, 100-calorie packages resulted in 25.2 % less consumption compared to larger, 400-calorie packages among college students [40]. Geier, Rozin, and Doros (2006) conducted three experiments in which they manipulated the participants by altering unit sizes (Tootsie Rolls, pretzels and M&M's). All the changes had significant effects on consumption as predicted: the consumed amount was substantially larger in the case of larger units than smaller units, as well as when the spoon was larger [26]. Similarly, Van Kleef et al. (2014) found that participants exposed to smaller unit sizes of chocolate bars consumed less overall, especially when the bars were unwrapped [22]. This suggests a psychological cue from the visibility of consumption. However, the impact of unit size is not uniform across all food types or contexts. Van Kleef et al. (2015) found that while portion size significantly influenced consumption among primary school children, unit size did not have a direct effect on intake when comparing whole cucumbers versus pre-sliced ones. The convenience of pre-sliced cucumbers did not alter children's perception of how much should be eaten, but rather affected consumption indirectly by making eating easier [41].

The interplay between unit size, packaging, and health consciousness is complex. The negative impact of larger unit sizes and the absence of packaging on food consumption and perceived energy intake is lessened among individuals with high levels of health consciousness. These individuals are more likely to engage in compensatory behaviours, such as consuming smaller portions in subsequent meals. Nevertheless, the protective effect of health consciousness is not absolute, indicating that interventions targeting environmental factors may be more effective in reducing overconsumption [35,36].

## 3 Material and methods

### 3.1 Participants

As part of the research, a total of four focus group surveys were conducted in two Hungarian cities, Pécs and Debrecen, in October 2019. Two separate groups were formed – one health-conscious and one not health-conscious (more of a flavor-conscious) group – in both cities, with the help of professional organizers. There were 8 participants in each group; thus, the experiment was carried out with 32 participants. The degree of health consciousness of the consumers was based on participants’ self-assessment, with the help of screening questionnaires.

The sociodemographic characteristics of the health-conscious groups were as follows: at least moderate income, no lower than secondary education, 4 participants under the age of 40, 4 participants over the age of 40, 3 males (either living alone or married) and 5 females (either living alone or married). The background of the non-health-conscious groups differed from that of the other group solely in the number of females and males (5 males, 3 females).

The focus group scenario was centred on the concept of nudging, which encompassed a number of key areas. These included purchase motivations, emotional map; the effect of unit size and packaging on the amount of food consumed (in the case of chocolate); consumer strategies in relation to the amount of food consumed; the impact of labels on taste perception (in the case of cheese). Consequently, the focus group survey included two distinct experiments. The findings of the “impact of labels on taste perception (in the case of cheese)" block have already been published by Szakály et al. (2020) [2]. The results of the block entitled ‘The effect of unit size and the presence of packaging on the amount of chocolate consumed’ are presented in this paper, among the listed topics/experiments.

All focus group discussions were always conducted by a professional moderator with relevant expertise in marketing and group dynamics and were conducted according to the guidelines laid down in the Declaration of Helsinki and all procedures involving human subjects were approved by the Research Ethics Committee at the University of Debrecen, Faculty of Economics and Business (approval number: GTKDH/75/2021). Verbal informed consent was obtained from all subjects. Verbal consent was witnessed and formally recorded.

### 3.2 Procedure

Prior to the commencement of the experiment, participants were provided with standardised instructions, which directed them to consume as much chocolate as they desired without any time constraints. To control for potential external influences on consumption, all participants were seated individually in separate spaces, thus minimising social influences or peer pressure. Additionally, each participant was provided with a refillable glass of mineral water, in order to standardise hydration, as thirst can influence consumption. The temperature of the room and lighting conditions were maintained at a constant level across all focus groups, in order to ensure a controlled environment, free from extraneous variables that might affect consumption behaviour.

In the experiment, each participant was given a specified amount of Kinder Bueno chocolate, which was available in two different sizes, either packed or unpacked. Each focus group (8 participants) was divided in four subgroups. Two participants in each focus group received three large units of packed chocolate, whereas two other participants got the same in an unpacked condition. The third subgroup of 2 people was given 13 small units of packed chocolate, while the last 2 people got the same in an unpackaged condition. The weight of the three large units of chocolate (66 g), and their energy content (369.5 kcal) was approximately the same as that of the 13 small units of Kinder Bueno (65 g, and 364 kcal). The participants were asked to eat as much chocolate as they wanted, and they felt like.

After eating the chocolate, participants were requested to estimate and record the amount they believed they had eaten, expressed in grams and calories. These estimates were subsequently entered into a database along with the actual consumption and energy intake data. The question was which variant would be most, and which the least, consumed by study participants.

### 3.3 Statistical analyses

In the course of the data analysis, several statistical methods were employed. The data were subjected to a three-way repeated measures ANOVA (RM-ANOVA) model during the analysis. In this experiment, the measurement time (before and after consumption) was identified as the within-subject effect, while health-conscious attitude, packaging and size were classified as the between-subject effects. One condition of the Repeated Measures ANOVA (RM-ANOVA) is that within each group the studied factors (perceived and real consumption in grams and calories) should be normally distributed, which could not be met in this case. Therefore, to assess the significance of the effect of the RM-ANOVA model, the Scheirer – Ray – Hare test was used instead of the F-probe. The procedure of the SRH test is described by Dytham (2011) [42]. The mean square (MS) should be considered from the RM-ANOVA model, which is the average of the squared differences between the observations and the grand mean. Then the sum of squared differences (SS) is calculated, as well as the SS/MS ratio for each factor. Based on these ratios, p-values can be computed according to a Chi-square distribution. In order to show differences between two related samples the Wilcoxon signed-rank test was applied. The significance level was 10 %, and all the calculations have been carried out using R Studio 4.0.0 [43]. A statistical post-hoc power analysis, and effect size calculations were performed using Superpower package in R 4.0.0. in order to measure the strength of relationships among the variables in the RM-ANOVA model. The statistical power is the probability of detecting an effect, while effect size measures the size of differences within the studied groups. The effect size (Cohen's f) in this study was considered small (0.1), medium (0.25) and large (0.4) using Cohen's (1969) criteria [44].

## 4 Results

The first step in the analysis was to assess how much of the chocolate distributed was consumed by the participants, separately by size and packaging. The results by unit size and packing variants are shown in Table 1.

**Table 1:** Real chocolate consumption by variants measured in weight (g) and energy (kcal) (*N* = 32).

Kinder Bueno chocolate bars					
Unit size	Packaging	Total weight (g)	Total energy (kcal)	Total consumption (g)	Total consumption (kcal)
Large	Packaged	528	2956	394	2208
	Unpackaged	528	2956	383	2145
Small	Packaged	520	2912	269	1504
	Unpackaged	520	2912	234	1312

As the table shows, the large units of chocolate available weighed 528 g altogether, equalling 2956 kcal (for 8 participants). The weight of the small units of chocolate available was only 8 g lower, which meant they contained 45 fewer calories. The results show that participants eating small units of chocolate generally consumed a third fewer calories, no matter whether it was packaged or unpackaged, compared to those who received large units. More specifically, it was unpackaged small units of chocolate that participants consumed the least of (29.3 g/person, that is 164 kcal), closely followed by packaged small units of chocolate (33.6 g/person, that is 188 kcal). As regards large units of chocolate, study subjects ate more of the packaged variant (49.3 g/person, that is 276.0 kcal), and a bit less of the unpackaged variant (47.9 g/person, that is 268.1 kcal/person).

After this, participants were asked to estimate how much chocolate they had consumed, both in grams and calories. The differences between estimates and real consumption are presented by product variants in Table 2.

**Table 2:** The real and perceived consumption of chocolate in terms of weight (g) and energy (kcal) (*N* = 32).

Kinder Bueno chocolate bars					
Unit size	Packaging	Real total consumption (g)	Perceived total consumption (g)	Real total consumption (kcal)	Perceived total consumption (kcal)
Large	Packaged	394	415	2208	2669
	Unpackaged	383	335	2145	1913
Small	Packaged	269	328	1504	2360
	Unpackaged	234	275	1312	2623

According to the results, consumers tended to overestimate the total amount of consumed chocolate, except for one variant (unpackaged large size). The most striking difference could be observed in the case of unpackaged small bars, where the estimate of total calorie consumption was double that of the real one (the difference in grams was only 17.5 %). This was followed by the category of packaged small units, where overestimation reached 56.9 % (overestimation in grams was 21.9 %). In terms of calories, packaged large bars occupied the third place, with an overestimation of 20.9 % (the overestimation in grams was 5.3 %). The case was exactly the opposite with unpackaged large products: calorie intake was underestimated by 12.1 %, which corresponded to −14.3 % in grams.

Table 3 presents the result of the Repeated Measure ANOVA analysis. In this model, repeated measurements occur over time, so time reflects the difference between real consumption (in grams and calories) and the consumption estimated after eating (expressed in grams and calories).

**Table 3:** Result of the Repeated Measure ANOVA analysis (*N* = 32).

Effect	Consumption in grams				Consumption in calories			
	Type III Sum of Squares	F-stat	SRH test	p-value	Type III Sum of Squares	F-stat	SRH test	p-value
*time*	*6340.14*	*3.57*	*4.76*	*0.017*	*142940.71*	*9.41*	*12.54*	*0.000*
*time∗health*	*9001.27*	*5.06*	*6.75*	*0.005*	9192.02	0.60	0.81	0.297
*time∗pack*	*3178.14*	*1.79*	*2.38*	*0.078*	150.68	0.01	0.01	0.908
*time∗size*	819.39	0.46	0.61	0.374	41077.16	*2.70*	*3.60*	*0.035*
*time∗health∗pack*	*11799.39*	*6.64*	*8.85*	*0.002*	1107.23	0.07	0.10	0.755
time∗health∗size	87.89	0.05	0.07	0.797	16224.39	1.07	1.42	0.164
*time∗pack∗size*	346.89	0.20	0.26	0.687	*77854.95*	*5.12*	*6.83*	*0.005*
time∗health∗ pack∗size	37.52	0.02	0.03	0.867	9905.23	0.65	0.87	0.277
Within subjects	42660.88				364742.0			
Total	42623.36				364742.0			
Mean Square	1333.15				11398.2			

As Table 3 shows, a significant difference could be detected between the real and perceived calorie consumption, both in grams and calories. The amount consumed expressed in grams was influenced both by healthy lifestyle and packaging (time∗health∗pack, p = 0.002), and healthy lifestyle alone also had an impact (time∗health, p = 0.005). In terms of calories, size and packaging together accounted for the differences (time∗pack∗size, p = 0.005), with size alone resulting in a difference between perceived and real calorie consumption (p = 0.035).

Table 4 gives a comprehensive account of the real and perceived amount and calorie content of chocolate consumed, separately by packaging and size.

**Table 4:** The amount and calorie content of chocolate consumed (by real and perceived consumption, separately by packaging and size) (*N* = 32).

Factor			Size						Total
			Small			Large			
			packaged	unpackaged	packaged + unpackaged	packaged	unpackaged	packaged + unpackaged	
Time	Real	grams^a^	33.6	29.3	31.4	49.3	47.9	48.5	40.3
		calories^b^	188.0	164.0	176.0	276.0	268.1	271.8	225.5
	Perceived	grams^a^	41.0	34.4	37.7	51.9	41.9	46.5	42.3
		calories^b^	295.0	327.9	311.4	333.6	239.1	283.2	296.8

a The correlation between the real and perceived consumption in grams is 0.493.

b The correlation between the real and perceived consumption in calories is 0.321.

As the model in Table 4 also reveals, in terms of calories it is the combination of size and packaging that influences consumers. The Wilcoxon test found no significant difference between packaged (Z = −1.183, p = 0.237) and unpackaged large units (Z = −0.560, p = 0.575), or between real and perceived calories either. Small units, however, produced significant differences between real and perceived calories both in the case of packaged (Z = −2.028, p = 0.043) and unpackaged products (Z = −2.197, p = 0.028). If only size is considered, small units still account for a significant difference: while real consumption was 176 calories, perceived consumption was estimated at 311.4 calories (Z = −2.983, p = 0.003).

**Fig. 1:**
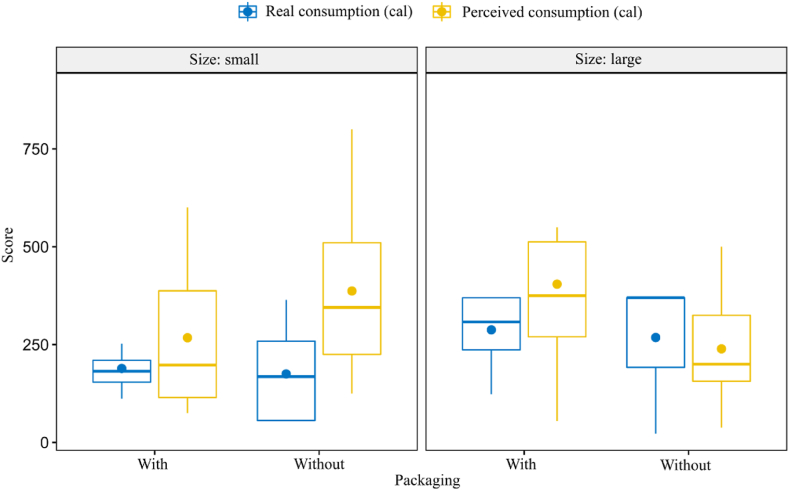
The amount and calorie content of the chocolate consumed (by real and perceived consumption, separately by packaging and size) (*N* = 32).

The differences between real and perceived calorie consumption are presented by size and packaging in Fig. 1. The figure clearly shows that the consumption of small units of chocolate led to a significant overestimation of the calories consumed, especially when the chocolate was unpackaged.

The next step was to explore the real and perceived amount and calorie content of chocolate consumed from a lifestyle perspective, taking into account the presence or absence of packaging. The results can be seen in Table 5.

**Table 5:** The amount and calorie content of the chocolate consumed (by real and perceived consumption, separately by packaging and healthy lifestyle) (*N* = 32).

Factor			Healthy lifestyle						Total
			yes			no			
			packaged	unpackaged	packaged + unpackaged	packaged	unpackaged	packaged + unpackaged	
Time	Real	grams^a^	32.5	26.4	31.7	48.1	53.9	50.4	40.3
		calories^b^	182.0	147.7	177.5	269.5	301.6	282.5	225.5
	Perceived	grams^a^	66.7	28.8	75.3	31.3	49.4	46.6	42.3
		calories^b^	200.8	223.5	248.0	399.4	345.7	400.9	296.8

a The correlation between the real and perceived consumption in grams is 0.493.

b The correlation between the real and perceived consumption in calories is 0.321.

As the model presented in Table 5 has also revealed, when consumption is viewed in grams, it is the combination of healthy lifestyle and packaging that influences consumers. The Wilcoxon test found a significant difference between real and perceived consumption in grams in the case of packaged products in the health-conscious group (Z = −2.366, p = 0.018) with no difference in the case of unpackaged chocolate (Z = −0.135, p = 0.893). If we only consider health-consciousness, the difference is still significant between the health-conscious and not health-conscious group. The real consumption in the health-conscious group was 31.7 g, whereas the perceived consumption was estimated at 75.3 g (Z = −2,197, p = 0.028).

**Fig. 2:**
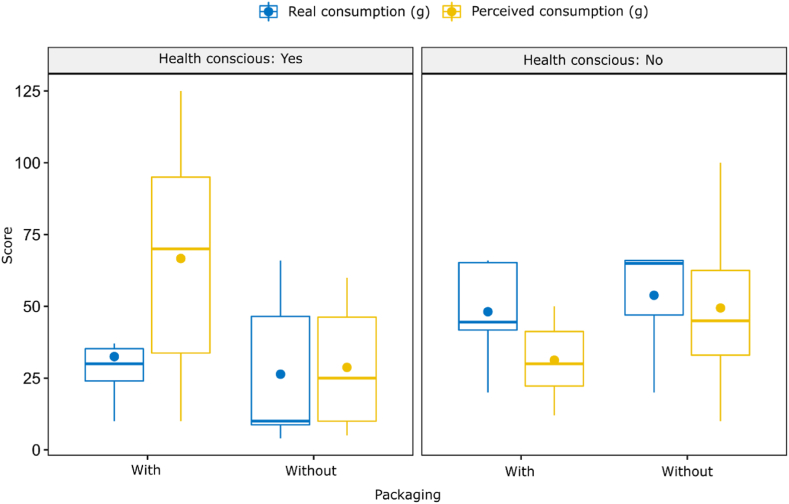
The amount and calorie content of the consumed chocolate (by real and perceived consumption, separately by packaging and healthy lifestyle) (*N* = 32).

The results of gram-based measurements of real and perceived consumption, shown by health-consciousness and packaging, are presented in Fig. 2, which accurately reflects that health-conscious consumers tended to most overestimate their consumption when the chocolate was packaged.

As the last step, a statistical post-hoc power analysis and effect size calculations were performed to measure the strength of relationships among the variables in the RM-ANOVA model. The statistical power is the probability of detecting an effect, while effect size measures the size of differences within the studied groups. Table 6 shows the result of the power analysis and effect size estimation.

**Table 6:** Result of the power analysis and effect size estimation (*N* = 32).

Effect	RM-ANOVA model for Consumption			
	in grams		in calories	
	Power	Effect size	Power	Effect size
time	20.4	0.222	68.7	0.600
size∗time			56.9	0.511
packaging∗time	25.5	0.273	10.5	0.046
health∗time	89.6	0.817		
size∗packaging∗time			25.7	0.275
health∗packaging∗time	69.3	0.604		

As Table 6 shows, the effects had enough power, especially the ‘health and time’ relationship and the ‘size and time’ relationship. Effect sizes are also large, indicating huge differences within the studied 8 groups, except for ‘packaging∗time’ and ‘size∗packaging∗time’, which only had a moderate effect. These results also indicate that the real and perceived consumption differs significantly, especially regarding size and health, and the interactions between the factors also play a significant role in consumption. The aforementioned power and Cohen's effect size calculations have demonstrated that the sample size is statistically sufficient for the conclusions drawn, and the research findings are practically significant.

## 5 Discussion

The results of the research study lead to the conclusion that consumption volume (expressed in grams or calories) is obviously influenced by unit size, and the presence or absence of packaging. The participants of each focus group consumed more of the product that came in a larger size, no matter if it was packaged or unpackaged, compared to smaller sizes. The difference between small and large unit variants of the same product was definitely more distinct compared to the difference seen between the packaged and unpackaged version of the same unit size. This clearly indicates that unit size has a much greater impact on consumption volume than packaging does. This, in turn, demonstrates the power of unit bias, which was already confirmed by previous research [22, 26, 37–39, 40, among others].

The volume of real consumption is probably closely linked to estimated (perceived) consumption, on a calorie basis: consumers ate the least of products the consumption of which they most overestimated. The finding was true in case of small unpackaged products, which is in line with the results of Van Kleef et al. (2014) [22]. This result alone is fairly important as non-use of packaging can provide essential support in promoting sustainable food consumption. Still, there is the question of why people tend to consume less chocolate when it comes in smaller units, even if in a definitely larger number? The power of the numerosity heuristic could also be felt here: a significant overestimation in both calories and grams indicates that study subjects considered the product to weigh more in the case of a higher number of small units (13 bars), even though the volume was the same as in the case of a small number of larger units [22, 27–29, 33, 34, 37–39, among others].

This points to the conclusion that consumers tend to rely more heavily on environmental cues rather than internal ones – the sensation of hunger in this case. Following the same logic, it is also clear that the more consumers overestimated their calorie consumption, the lower the real consumption was. The variant that came in a large size and was unpackaged also fits this logic: in this case consumers underestimated their calorie consumption, consequently they consumed more of this product without feeling any guilt, as they were not aware of their real calorie intake.

Conclusions about health-conscious lifestyles and the role of packaging in consumption can also be drawn from the research. People who considered themselves health-conscious consumed remarkably fewer packaged products, and tended to greatly overestimate the amount they had consumed. This indicates that those who are health-conscious may be more aware of their dietary intake, which may result in an overestimation of the amount consumed. At the same time, non health-conscious consumers ate more than they estimated, regardless of whether the product was packaged or not. It is interesting that considering health consciousness, this significant difference in calories could only be observed in the case of packaged products. The effort related to unpacking the product as well as empty wrapping papers (as external cues) gave a warning signal at an earlier point for health conscious consumers [31,35,36,40]. In addition, as they were tasting hedonic products, non health-conscious consumers were focusing mainly on taste and hedonic benefits [25].

When viewed in the context of previous research on food consumption and the effects of labelling, this study reveals significant divergences. A review of the literature reveals that studies examining factors such as nutritional labelling, health warnings, or calorie content display have typically concluded that labels influence consumers' conscious decision-making [[45, 46, 47, 48, 49, 50], among others]. This is because the availability of information increases awareness and facilitates more deliberate food choices. However, the present study does not focus on conscious decision-making, but rather on decisions made outside of conscious control.

While the effects of labelling operate at the cognitive level, influencing consumers to interpret information and make deliberate choices, unit size functions primarily at the behavioural level. Modifying unit size requires no particular cognitive effort or conscious decision on the part of the consumer; it directly affects the amount consumed, leading to greater consumption with larger portions.

Furthermore, the variable of packaging differs significantly from that of labeling effects, as evidenced by previous studies. In contrast to research on product labelling, which is concerned with the presentation of information, the function of packaging is to introduce mechanical barriers that encourage consumers to be more mindful of the quantity they are consuming [11]. The act of consuming an item that has been individually packaged prompts the consumer to pause and reflect on the quantity they are consuming. In contrast, larger, unpackaged portions tend to result in automatic, greater consumption.

Therefore, the findings of this study indicate that while studies focusing on labelling [11] have demonstrated that cognitive nudges can influence consumer awareness and perception of food choices, this study demonstrates that unit size operates as a more powerful behavioural nudge. In contrast to labelling, which alters consumer perceptions, unit size directly impacts consumption through automatic, unconscious decision-making processes.

## 6 Conclusion

The experiment yielded instructive results regarding the influence of unit size and packaging on chocolate consumption. Through the analysis of actual and perceived consumption, a number of key conclusions can be drawn, which highlight the complex interaction between these variables and their impact on eating behaviour.

The final conclusion that can be drawn from the results is that external cues have a major impact on behavior even when consumers’ level of knowledge or emotions are not altered or influenced. These results demonstrate the pivotal influence of unit size and packaging on food consumption, in alignment with previous studies indicating that larger unit sizes lead to higher consumption. In the experiment, unit size had a greater effect on consumption than packaging. The psychological effect of smaller unit sizes is well illustrated by the difference between actual and perceived consumption, as participants generally overestimated the amount of chocolate they consumed. The overestimation was particularly striking for small, unwrapped units, where participants believed they had consumed twice as much as they actually did.

Examining the role of healthy lifestyle, the study found that health-conscious participants exhibited a greater tendency to overestimate their consumption, especially of packaged chocolates. This suggests that health-conscious individuals may be more conscious of their eating habits, yet may still misjudge the amount consumed, especially if the food is packaged. Increased vigilance or anxiety about food may contribute to this overestimation.

The statistical analysis revealed an interaction between the factors studied. A significant difference was observed between actual and perceived calorie consumption, which was mostly influenced by unit size and packaging. While health consciousness was the most important factor affecting consumption and estimates in grams. The interactions demonstrated the complex nature of eating behaviour, where both physical and psychological factors combine to influence overall intake. The significant differences between actual and perceived consumption also highlight the need for more nuanced approaches to food presentation and portion control, particularly in contexts prone to overeating.

In conclusion, the results of the research provide evidence that behavioural nudge marketing tools are an effective method of influencing consumer behaviour. Such a tool proved to be highly effective in achieving more sustainable and also healthier consumption among consumers in this case study. An understanding of this mechanism can inform the development of strategies for the regulation of food intake and the adoption of healthier eating behaviours.

The results of the study may have broader applications beyond the specific study of chocolate consumption in the field of nudge theory. Nudge theory is based on the idea that small changes in the decision-making environment can influence people's behaviour without forcing them or changing their core knowledge. Interventions of this type are often based on small environmental changes that effectively influence people's choices without them being aware of the manipulation. The results of this study, which explores the effects of unit size and packaging on chocolate consumption, fit well with these principles of nudge theory.

The effectiveness of unit size reduction is not limited to chocolate, but can be applied to other types of food and products. For example, in the case of fast food, snacks or even soft drinks, smaller units and packaging can similarly reduce overconsumption, which can help to promote healthier eating habits. In addition, these findings support public health strategies that aim to reduce the consumption of high-calorie foods and beverages by reducing their size without people being aware of the change.

The study results also support the idea that packaging can be an effective nudge tool. Individually packaged smaller units can effectively control consumption not only of confectionery but also of other foods. This packaging strategy, which raises consumer awareness by fragmenting decisions, encourages consumers to pause and think about their choices. For example, packaging snacks or meals so that each portion is individually wrapped can help reduce impulse overconsumption. The application of nudge theory can also extend to promoting environmentally responsible behaviour. For example, the introduction of smaller packaging units can encourage less consumption and therefore less waste. Packaging, as a visual and practical tool, can encourage people to consume less and make more conscious choices to protect the environment.

The results suggest that nudge strategies around unit size and packaging can be applied not only in the food industry but also in other sectors. The introduction of smaller units in school catering, hospitals or other public institutions can help reduce calorie consumption and contribute to the fight against obesity.

The results of the study provide valuable insights into the practical application of nudge theory, in particular by examining the effects of unit size and packaging. The findings have wider applications in the food industry, public health, the environment and other areas where influencing consumption patterns is important. The theoretical and practical relevance of nudge theory in the light of these findings can be extended by further research, in particular by investigating the long-term effects of these strategies on consumer behaviour.

### 6.1 Limitations and recommendations

While the experiment presented in this study has yielded valuable results, it is important to consider the limitations of the findings when evaluating them.

The sample used in the study is not representative, which limits the generalisability of the results. The characteristics of the individuals who participated in the study do not reflect the demographic composition of Hungarian society. This is due to two factors: the relatively small sample size (N = 32) and the fact that the participants were not selected randomly. The participants were selected via screening questionnaires and included in the experiment on the basis of predefined criteria. The study population was selected based on the socio-demographic characteristics of the participants, including age, gender, income status, and health status. Individuals with at least a secondary education and a moderate income were included in the study. This consequently excludes social groups with lower levels of education or income, as their consumption patterns may differ from those observed in this study. Moreover, the study was conducted in only two cities, which limits the generalisability of the findings to the Hungarian population as a whole, given the country's geographical, cultural and social diversity. It would be beneficial for future research to randomly select a larger, more representative sample with a greater number of focus groups in order to more accurately reflect the actual consumption patterns of Hungarian society and to generalise the results to other social strata and regions. It is important to note, however, that this exploratory pilot study with a relatively small sample size was primarily designed to test the methodology. The objective of presenting quantitative data and its statistical analysis was to elucidate the existence of differences. As the methodology of the study has been demonstrated to be effective in demonstrating differences, it may also be employed in the presentation of a representative sample.

The experiment did not take into account psychological factors, which may restrict the ability to draw more profound conclusions from the results. It is possible that personality traits, such as impulsivity, mindfulness, or stress responses, may exert a significant influence on eating behaviour, which the current study did not measure. It may be beneficial to ascertain the personality type of each participant in order to gain insight into how these factors influence their behaviour during the experiment. It is recommended that future studies include the input of a psychologist in order to identify and analyse different personality types. This could facilitate a more nuanced understanding of the influence of specific personality traits on consumption patterns and the efficacy of nudging strategies across diverse psychological profiles.

A further limitation of the present study is that, although the results and literature suggest that larger unit sizes tend to increase consumption, particularly for product such as chocolate, it is important to consider that this effect can vary significantly depending on the type of food and its presentation. The consumption of indulgence products (e.g. sweets, snacks) is associated with a reduction in self-control, particularly when the food is served in larger units. This may be in contrast to the consumption of healthier foods (e.g. fruit, vegetables), where larger units may have less influence on consumption patterns. Furthermore, the manner in which the food is presented, including the packaging and serving, may also influence the impact of unit size on consumption. The results of the study demonstrated that the combination of packaging and unit size had a significant effect on the consumption of chocolate. However, the presentation of other foods, such as the appearance or layout of a dish, may have disparate effects on consumption.

It is also important to note that the controlled nature of the artificial experimental environment differed significantly from the variety of consumption situations encountered in real life, which introduced another limitation to the study. The consumption of sweets by participants in a focus group was observed under specific conditions, which does not provide an accurate representation of the typical circumstances under which individuals make food consumption decisions. These decisions are often influenced by a range of emotional, social, and spontaneous factors. In real life, a multitude of additional factors and roles must be considered, including heuristics that facilitate decision-making, advertising, and social interactions. Furthermore, in circumstances where an individual's dietary habits are not subject to conscious reflection, emotional states such as stress, happiness or even fatigue have the potential to exert a considerable influence on consumption patterns (see emotional eating). Nevertheless, in a controlled experiment, these factors can be excluded, thereby providing a limited and less realistic representation of actual behaviour. It is recommended that future research should endeavour to model situations that are closer to real-life consumption situations. Such experiments could be conducted in the field, with participants receiving the product in their home environment or at work. Furthermore, it may prove beneficial to extend the experiment to encompass additional factors, such as emotional state or peer interactions. This could potentially yield valuable new data for a more comprehensive understanding of consumption patterns.

The short-term nature of the research is indicated by the fact that the experiment examined a one-off consumption situation, which precludes any consideration of long-term effects. The efficacy of behavioural nudges, such as altering unit size, may diminish over time. This is because participants' initial responses to smaller unit sizes would likely habituate over time, leading to altered consumption patterns in the longer term. Future research conducted over a longer time period may help to elucidate whether the effects of unit size can be sustained and how people adapt to changing portion sizes in the long term. For example, a longitudinal study monitoring participants' chocolate consumption over months could provide valuable insights.

The experiment must account for additional biases. A potential source of bias in the experiment was the awareness of the participants that they were being observed, which may have influenced their behaviour. It is possible that participants exhibited a ‘compliance’ or ‘expectation’ compulsion, whereby they behaved in accordance with the expectations of the researchers. Conversely, it has been demonstrated that individuals consume less food when they are aware that they are being observed.

It is also possible that individual characteristics and situational factors of the participants in the experiment may have exerted a biasing effect. The results may have been influenced by individual characteristics, such as general preferences for chocolate and specific brand preferences for Kinder Bueno. The influence of individual eating behaviours, including intuitive eating, emotional eating, restrictive/dietary eating and unrestricted eating, on the results may have been significant. Furthermore, the experiment did not account for individual perceptions of hunger as a situational factor. In the future, a longer-term observational study of participants' behaviour in their natural environment may help to elucidate the extent to which behavioural modification is maintained over time.

These experimental limitations may have important implications for the generalisability and accuracy of the results. Although the experiment yielded valuable results, it would be beneficial for future research to take these limitations into account, for example by including larger and more representative samples, using random selection, and analysing psychological factors, in order to improve the reliability and validity of studies.

### 6.2 Implications

The findings of the article present a series of pragmatic recommendations that can assist food manufacturers and policymakers in fostering healthier dietary patterns. Those engaged in the formulation of public policy may utilize the findings to inform the development of regulations or guidelines pertaining to unit sizes, particularly within the context of public institutions such as schools or hospitals. The utilisation of smaller units in such institutions may assist in the promotion of more moderate eating habits and the prevention of obesity.

The results of this study could inform the development of public health campaigns aimed at reducing obesity rates, in particular those that target the consumption of high-calorie foods. It may be possible for policymakers to utilize packaging regulations in order to guarantee that foods which are high in fat or sugar are made available in smaller, more manageable unit sizes. It would be beneficial for policymakers to implement policies and nutrition campaigns that raise awareness of the importance of unit sizes and the impact of packaging on daily eating habits. Information campaigns could place an emphasis on the advantages of smaller units and more conscious consumption.

Additionally, manufacturers could market products in smaller units, with packaging that serves a dual purpose of facilitating consumption while also enabling informed consumer choices. In the case of individually wrapped confectionery, for example, opening each pack requires a conscious decision, which can prevent automatic, impulsive overconsumption. Smaller units can also be positioned as health-conscious alternatives that allow consumers to enjoy their favourite products without overconsumption. Such marketing strategies can encourage people to make more informed choices when consuming food.

## CRediT authorship contribution statement

**Enikő Kontor:** Writing – review & editing, Writing – original draft, Methodology, Investigation, Conceptualization. **Mihály Soós:** Writing – original draft, Methodology, Investigation, Conceptualization. **Nikolett Balsa-Budai:** Writing – original draft, Methodology, Investigation, Formal analysis, Conceptualization. **Sándor Kovács:** Writing – original draft, Methodology, Formal analysis, Conceptualization. **Zoltán Szakály:** Writing – review & editing, Writing – original draft, Methodology, Investigation, Conceptualization.

## Ethical statement

The study obtained ethical approval (approval number: GTKDH/75/2021) from the Research Ethics Committee of the University of Debrecen, Faculty of Economics and Business.

Participants gave informed consent before taking part in the research. Verbal consent was audio-recorded and the recording was approved by the participants. Verbal consent is an accepted method in consumer behaviour research.

## Data availability

Mendeley Data, V1, https://doi.org/10.17632/ptt8zxzx92.1.

## Declaration of competing interest

The authors declare that they have no known competing financial interests or personal relationships that could have appeared to influence the work reported in this paper.
