# Supplementary material for: The unit size effect on chocolate consumption: How to make consumers eat less? (The unit size effect on chocolate consumption)
Source: Heliyon. 2024 Dec 13;11(1):e41139. doi: 10.1016/j.heliyon.2024.e41139 (PMC11719318; doi:10.1016/j.heliyon.2024.e41139)
Supplement: Multimedia component 1 [file mmc1.docx]

**SCRIPT (MODERATOR GUIDE)**

**Analysis of food preferences and emotions**

THANK PARTICIPANTS, INTRODUCE YOURSELF! BRIEFLY EXPLAIN WHAT WILL HAPPEN AND WHY THEY ARE HERE! EMPHASIZE THE IMPORTANCE OF HONEST RESPONSES! AGREE ON WHETHER TO SPEAK UP OR RAISE HANDS DURING THE DISCUSSION! ASK IF ANYONE HAS ANY QUESTIONS OR COMMENTS?

**1. Participant Introductions (5 minutes)**

First, I would like to ask each of you to *briefly introduce yourselves*! How old are you, what do you do for a living, and how do you usually spend your free time? If there's anything else you feel is important to share about yourselves, we'd love to hear it. MAKE SURE EVERYONE GETS A CHANCE TO SPEAK, PAY SPECIAL ATTENTION TO THE SHY ONES!

**2. Association Game (10 minutes)**

Thank you for the introductions! As a warm-up, I'd like to play an association game with you. I'll say a word, and I'd like you to say the first thing that comes to mind without overthinking it. It could be a word, a phrase, or anything else that pops into your head. We'll go one by one, starting with XY! HERE WE'RE LOOKING FOR WHETHER THE ASSOCIATION WITH THE WORD IS NEGATIVE OR POSITIVE, AND WHETHER IT'S RATIONAL OR EMOTIONAL.

*chocolate, cheese, low-fat content, Dots (brand), low sugar content, milk, Coca-Cola, pork, Danone, poultry, low salt content, Heart-Healthy label, Hungarian product, Nestlé, yogurt, Szentkirályi (brand), oatmeal, fruit, potato chips*

**3. Shopping Motivations, Emotional Mapping (30 minutes)**

When you go shopping, what factors do you consider when making choices? Let's take examples! You want to buy chocolate. Mention up to 5 criteria! Yogurt. Potato chips. Sausage. **Now I'll hand out a short questionnaire**. Please fill it out regarding **chocolate**! First, imagine eating a chocolate bar! What are your strongest thoughts (opinions, prejudices, emotions, etc.) regarding eating chocolate? And the weakest? Now, let's actually taste some chocolate. After that, **please fill out another questionnaire**! How do you feel now? Has your opinion changed in any way? If so, how? Let's discuss the opinions. How do you think the results could be used in product development?

**4. The Effect of Unit Size on Food Consumption (15 minutes)**

As the next task, we'll divide participants into four groups. Everyone will receive a certain amount of Kinder Bueno chocolate, available in two sizes, packaged and unpackaged. Eat as much as you want or feel comfortable with. We'll distribute the samples. Estimate how much chocolate you've eaten in grams. And in calories? **Write it down on a piece of paper!** Which size do you prefer, smaller or larger? Which encourages more consumption (per unit size)? Do you think packaged or unpackaged chocolate encourages more consumption?

**5. Consumer Strategies Regarding Food Consumption (15 minutes)**

How consciously do you decide what foods to buy? How consciously do you decide how much of the purchased foods to eat? Which is the easier decision: selecting foods that are right for you (e.g., healthy, tasty) or eating less of these foods? Can you give good examples? And bad examples? How many decisions about food and drink do you make in a day? How many thoughts do you have about eating in a day? Describe a typical shopping process from start to finish (*making a list, impulse buying, buying larger portions, buying tempting products, enjoying tastings in stores*)! Now, a typical dinner *(how often you buy, select foods for the table, don't put everything in the fridge, avoid tempting foods*)! Now, a typical restaurant meal (*motivations, don't eat everything on the plate, consciously limit quantity, no appetizers and desserts, stop eating when full, leave food leftovers if too much*)! In what situations do you eat more than usual? Write down such cases! Do you feel guilty then? Does the quantity influence the societal consumption norm, the group effect? Does it matter if you eat in a group or alone? What are the reasons for overconsumption (overeating)?

**6. The effect of labels on taste perception (20 minutes)**

Finally, we distribute 4 samples of semi-hard matured cheese to everyone, with a different composition and with different inscriptions (labels). Samples include regular store-bought cheese, reduced-salt cheese, reduced-fat cheese, reduced-fat and reduced-salt cheese. I will hand out a test sheet for preliminary (before tasting) testing. Let's discuss the results! I am now handing out another test sheet for post-tasting testing. Let's discuss the results!

**Conclusion, closing, gift-giving.**

**Test sheet I.**

**Below is a list of words describing emotions and moods. Using these, describe: how do you feel when you imagine eating chocolate?**

**1 – Not feeling at all 5 – Feeling completely**

| **Emotional characteristic** | **Grade** |
| --- | --- |
| Happy | 1 – 2 – 3 – 4 – 5 |
| Wild | 1 – 2 – 3 – 4 – 5 |
| Content | 1 – 2 – 3 – 4 – 5 |
| Pleasant | 1 – 2 – 3 – 4 – 5 |
| Guilty | 1 – 2 – 3 – 4 – 5 |
| Friendly | 1 – 2 – 3 – 4 – 5 |
| Lovely | 1 – 2 – 3 – 4 – 5 |
| Energetic | 1 – 2 – 3 – 4 – 5 |
| Active | 1 – 2 – 3 – 4 – 5 |
| Heart-warming | 1 – 2 – 3 – 4 – 5 |
| Soothing | 1 – 2 – 3 – 4 – 5 |
| Delightful | 1 – 2 – 3 – 4 – 5 |
| Enthusiastic | 1 – 2 – 3 – 4 – 5 |
| Peaceful | 1 – 2 – 3 – 4 – 5 |
| Nostalgic | 1 – 2 – 3 – 4 – 5 |
| Troubled | 1 – 2 – 3 – 4 – 5 |
| Safe | 1 – 2 – 3 – 4 – 5 |
| Cheerful | 1 – 2 – 3 – 4 – 5 |
| Adventurous | 1 – 2 – 3 – 4 – 5 |
| Free | 1 – 2 – 3 – 4 – 5 |
| In love | 1 – 2 – 3 – 4 – 5 |
| Interested | 1 – 2 – 3 – 4 – 5 |
| Disgusting | 1 – 2 – 3 – 4 – 5 |
| Balanced | 1 – 2 – 3 – 4 – 5 |
| Quiet | 1 – 2 – 3 – 4 – 5 |
| Gentle | 1 – 2 – 3 – 4 – 5 |
| Excited | 1 – 2 – 3 – 4 – 5 |
| Light | 1 – 2 – 3 – 4 – 5 |
| Polite | 1 – 2 – 3 – 4 – 5 |
| Understanding | 1 – 2 – 3 – 4 – 5 |
| Boring | 1 – 2 – 3 – 4 – 5 |
| Sensitive | 1 – 2 – 3 – 4 – 5 |
| Brave | 1 – 2 – 3 – 4 – 5 |
| Aggressive | 1 – 2 – 3 – 4 – 5 |
| Cheerful | 1 – 2 – 3 – 4 – 5 |
| Good | 1 – 2 – 3 – 4 – 5 |

**Test sheet II.**

**Below is the same list of words referring to emotions as in the previous questionnaire. Using these, describe: how do you feel now, having actually consumed the chocolate?**

**1 – Not feeling at all 5 – Feeling completely**

| **Emotional characteristic** | **Grade** |
| --- | --- |
| Happy | 1 – 2 – 3 – 4 – 5 |
| Wild | 1 – 2 – 3 – 4 – 5 |
| Content | 1 – 2 – 3 – 4 – 5 |
| Pleasant | 1 – 2 – 3 – 4 – 5 |
| Guilty | 1 – 2 – 3 – 4 – 5 |
| Friendly | 1 – 2 – 3 – 4 – 5 |
| Lovely | 1 – 2 – 3 – 4 – 5 |
| Energetic | 1 – 2 – 3 – 4 – 5 |
| Active | 1 – 2 – 3 – 4 – 5 |
| Heart-warming | 1 – 2 – 3 – 4 – 5 |
| Soothing | 1 – 2 – 3 – 4 – 5 |
| Delightful | 1 – 2 – 3 – 4 – 5 |
| Enthusiastic | 1 – 2 – 3 – 4 – 5 |
| Peaceful | 1 – 2 – 3 – 4 – 5 |
| Nostalgic | 1 – 2 – 3 – 4 – 5 |
| Troubled | 1 – 2 – 3 – 4 – 5 |
| Safe | 1 – 2 – 3 – 4 – 5 |
| Cheerful | 1 – 2 – 3 – 4 – 5 |
| Adventurous | 1 – 2 – 3 – 4 – 5 |
| Free | 1 – 2 – 3 – 4 – 5 |
| In love | 1 – 2 – 3 – 4 – 5 |
| Interested | 1 – 2 – 3 – 4 – 5 |
| Disgusting | 1 – 2 – 3 – 4 – 5 |
| Balanced | 1 – 2 – 3 – 4 – 5 |
| Quiet | 1 – 2 – 3 – 4 – 5 |
| Gentle | 1 – 2 – 3 – 4 – 5 |
| Excited | 1 – 2 – 3 – 4 – 5 |
| Light | 1 – 2 – 3 – 4 – 5 |
| Polite | 1 – 2 – 3 – 4 – 5 |
| Understanding | 1 – 2 – 3 – 4 – 5 |
| Boring | 1 – 2 – 3 – 4 – 5 |
| Sensitive | 1 – 2 – 3 – 4 – 5 |
| Brave | 1 – 2 – 3 – 4 – 5 |
| Aggressive | 1 – 2 – 3 – 4 – 5 |
| Cheerful | 1 – 2 – 3 – 4 – 5 |
| Good | 1 – 2 – 3 – 4 – 5 |

Before tasting, tell us what is your first association generated by each version of the product?

**Test Sheet III.**

1. **Before tasting, to what extent do you think you will like them?**

*Conventional cheese*

| Extremely dislike |  |  |  |  |  | Extremely like |
| --- | --- | --- | --- | --- | --- | --- |
| 1 | 2 | 3 | 4 | 5 | 6 | 7 |

*Reduced-sodium cheese*

| Extremely dislike |  |  |  |  |  | Extremely like |
| --- | --- | --- | --- | --- | --- | --- |
| 1 | 2 | 3 | 4 | 5 | 6 | 7 |

*Reduced-fat cheese*

| Extremely dislike |  |  |  |  |  | Extremely like |
| --- | --- | --- | --- | --- | --- | --- |
| 1 | 2 | 3 | 4 | 5 | 6 | 7 |

*Reduced fat- and reduced-sodium cheese*

| Extremely dislike |  |  |  |  |  | Extremely like |
| --- | --- | --- | --- | --- | --- | --- |
| 1 | 2 | 3 | 4 | 5 | 6 | 7 |

1. **Before tasting, to what extent do you think each version of the product will be salty?**

*Conventional cheese*

| Not salty  at all |  |  |  |  |  | Extremely salty |
| --- | --- | --- | --- | --- | --- | --- |
| 1 | 2 | 3 | 4 | 5 | 6 | 7 |

*Reduced-sodium cheese*

| Not salty  at all |  |  |  |  |  | Extremely salty |
| --- | --- | --- | --- | --- | --- | --- |
| 1 | 2 | 3 | 4 | 5 | 6 | 7 |

*Reduced-fat cheese*

| Not salty  at all |  |  |  |  |  | Extremely salty |
| --- | --- | --- | --- | --- | --- | --- |
| 1 | 2 | 3 | 4 | 5 | 6 | 7 |

*Reduced fat- and reduced-sodium cheese*

| Not salty  at all |  |  |  |  |  | Extremely salty |
| --- | --- | --- | --- | --- | --- | --- |
| 1 | 2 | 3 | 4 | 5 | 6 | 7 |

1. **Before tasting, to what extent do you think each version of the product will be fatty?**

*Conventional cheese*

| Not fatty at all |  |  |  |  |  | Extremely fatty |
| --- | --- | --- | --- | --- | --- | --- |
| 1 | 2 | 3 | 4 | 5 | 6 | 7 |

*Reduced-sodium cheese*

| Not fatty at all |  |  |  |  |  | Extremely fatty |
| --- | --- | --- | --- | --- | --- | --- |
| 1 | 2 | 3 | 4 | 5 | 6 | 7 |

*Reduced-fat cheese*

| Not fatty at all |  |  |  |  |  | Extremely fatty |
| --- | --- | --- | --- | --- | --- | --- |
| 1 | 2 | 3 | 4 | 5 | 6 | 7 |

*Reduced fat- and reduced-sodium cheese*

| Not fatty at all |  |  |  |  |  | Extremely fatty |
| --- | --- | --- | --- | --- | --- | --- |
| 1 | 2 | 3 | 4 | 5 | 6 | 7 |

1. **Before tasting, to what extent do you think you will be willing to purchase each version of the product?**

*Conventional cheese*

| Not willing to purchase at all |  |  |  |  |  | Extremely willing to purchase |
| --- | --- | --- | --- | --- | --- | --- |
| 1 | 2 | 3 | 4 | 5 | 6 | 7 |

*Reduced-sodium cheese*

| Not willing to purchase at all |  |  |  |  |  | Extremely willing to purchase |
| --- | --- | --- | --- | --- | --- | --- |
| 1 | 2 | 3 | 4 | 5 | 6 | 7 |

*Reduced-fat cheese*

| Not willing to purchase at all |  |  |  |  |  | Extremely willing to purchase |
| --- | --- | --- | --- | --- | --- | --- |
| 1 | 2 | 3 | 4 | 5 | 6 | 7 |

*Reduced fat- and reduced-sodium cheese*

| Not willing to purchase at all |  |  |  |  |  | Extremely willing to purchase |
| --- | --- | --- | --- | --- | --- | --- |
| 1 | 2 | 3 | 4 | 5 | 6 | 7 |

**Test Sheet IV.**

1. **After tasting, how much do you like each version of the product?**

*Conventional cheese*

| Extremely dislike |  |  |  |  |  | Extremely like |
| --- | --- | --- | --- | --- | --- | --- |
| 1 | 2 | 3 | 4 | 5 | 6 | 7 |

*Reduced-sodium cheese*

| Extremely dislike |  |  |  |  |  | Extremely like |
| --- | --- | --- | --- | --- | --- | --- |
| 1 | 2 | 3 | 4 | 5 | 6 | 7 |

*Reduced-fat cheese*

| Extremely dislike |  |  |  |  |  | Extremely like |
| --- | --- | --- | --- | --- | --- | --- |
| 1 | 2 | 3 | 4 | 5 | 6 | 7 |

*Reduced fat- and reduced-sodium cheese*

| Extremely dislike |  |  |  |  |  | Extremely like |
| --- | --- | --- | --- | --- | --- | --- |
| 1 | 2 | 3 | 4 | 5 | 6 | 7 |

1. **After tasting, to what extent do you find each version of the product salty?**

*Conventional cheese*

| Not salty  at all |  |  |  |  |  | Extremely salty |
| --- | --- | --- | --- | --- | --- | --- |
| 1 | 2 | 3 | 4 | 5 | 6 | 7 |

*Reduced-sodium cheese*

| Not salty  at all |  |  |  |  |  | Extremely salty |
| --- | --- | --- | --- | --- | --- | --- |
| 1 | 2 | 3 | 4 | 5 | 6 | 7 |

*Reduced-fat cheese*

| Not salty  at all |  |  |  |  |  | Extremely salty |
| --- | --- | --- | --- | --- | --- | --- |
| 1 | 2 | 3 | 4 | 5 | 6 | 7 |

*Reduced fat- and reduced-sodium cheese*

| Not salty  at all |  |  |  |  |  | Extremely salty |
| --- | --- | --- | --- | --- | --- | --- |
| 1 | 2 | 3 | 4 | 5 | 6 | 7 |

1. **After tasting, to what extent do you find each version of the product fatty?**

*Conventional cheese*

| Not fatty  at all |  |  |  |  |  | Extremely fatty |
| --- | --- | --- | --- | --- | --- | --- |
| 1 | 2 | 3 | 4 | 5 | 6 | 7 |

*Reduced-sodium cheese*

| Not fatty  at all |  |  |  |  |  | Extremely fatty |
| --- | --- | --- | --- | --- | --- | --- |
| 1 | 2 | 3 | 4 | 5 | 6 | 7 |

*Reduced-fat cheese*

| Not fatty  at all |  |  |  |  |  | Extremely fatty |
| --- | --- | --- | --- | --- | --- | --- |
| 1 | 2 | 3 | 4 | 5 | 6 | 7 |

*Reduced fat- and reduced-sodium cheese*

| Not fatty  at all |  |  |  |  |  | Extremely fatty |
| --- | --- | --- | --- | --- | --- | --- |
| 1 | 2 | 3 | 4 | 5 | 6 | 7 |

1. **After tasting, to what extent would you be willing to purchase each version of the product?**

*Conventional cheese*

| Not willing to purchase at all |  |  |  |  |  | Extremely willing to purchase |
| --- | --- | --- | --- | --- | --- | --- |
| 1 | 2 | 3 | 4 | 5 | 6 | 7 |

*Reduced-sodium cheese*

| Not willing to purchase at all |  |  |  |  |  | Extremely willing to purchase |
| --- | --- | --- | --- | --- | --- | --- |
| 1 | 2 | 3 | 4 | 5 | 6 | 7 |

*Reduced-fat cheese*

| Not willing to purchase at all |  |  |  |  |  | Extremely willing to purchase |
| --- | --- | --- | --- | --- | --- | --- |
| 1 | 2 | 3 | 4 | 5 | 6 | 7 |

*Reduced fat- and reduced-sodium cheese*

| Not willing to purchase at all |  |  |  |  |  | Extremely willing to purchase |
| --- | --- | --- | --- | --- | --- | --- |
| 1 | 2 | 3 | 4 | 5 | 6 | 7 |
